# Integrated gut microbiota and metabolomic analysis reveals immunomodulatory effects of Echinacea extract and Astragalus polysaccharides

**DOI:** 10.3389/fvets.2022.971058

**Published:** 2022-09-02

**Authors:** Shaochuan Li, Renzhao Lin, Jiaxin Chen, Riaz Hussain, Shiwei Zhang, Yalin Su, Yanzi Chan, Abdul Ghaffar, Dayou Shi

**Affiliations:** ^1^College of Veterinary Medicine, South China Agricultural University, Guangzhou, China; ^2^The Islamia University of Bahawalpur, Bahawalpur, Pakistan

**Keywords:** gut microbiota, metabolomic analysis, immunosuppression, Echinacea extract, Astragalus polysaccharides

## Abstract

Immunosuppression in different animals increases the susceptibility of various infections caused by pathogenic microorganisms leading to increase risks posed by antibiotics in different animal farming sectors. Therefore, investigation of the interactions between natural medicines and the intestinal environmental ecosystem is of vital importance and crucial. This study for the first time investigated the effects of Echinacea Extract (EE) and Astragalus polysaccharide (APS) on the gut using 16S rRNA and metabolomic analysis approaches in immunosuppressed broiler chickens. There were four groups divided into control (C), immunosuppression (IS), EE, and APS groups. Sequencing of gut microbes showed that immunosuppression decreased the relative abundance of *Anaerofustis, Anaeroplasma, Anaerotroncus*, and *Lachnospira* in the gut while increasing that of *c_115* and *Holdemania*. However, EE and APS diminished the effects on the immunosuppression on the microbiota. The results revealed up-regulation of the relative abundance of *Enterococcus* in broiler chickens. In addition, EE reduced the relative abundance of *Ruminococcus* and *Blautia*. The results on metabolomic analysis revealed that immunosuppression mainly affects cyanuric acid metabolism, starch and sucrose metabolism while interconversion of pentose and glucuronide. EE and APS, on the other hand mainly impact butyrate metabolism, alanine, aspartate and glutamate metabolism while the interconversion of pentose and glucuronide, and D-glutamine and D-glutamate metabolism. Results regarding correlation analysis revealed significantly metabolic pathways including TCA cycle, butyrate metabolism, glyoxylate and dicarboxylate metabolism, propionate metabolism, alanine, aspartate and glutamate metabolism associated with *Ruminococcus* and *Blautia*. Both EE and APS can antagonize the effects of immunosuppression by modulating the disrupted gut microbiota. Nevertheless, EE might have a bidirectional regulatory functions on the intestinal health and further studies are needed to know the exact and relevant mechanisms of action regarding the effects of EE and APS.

## Introduction

Immunosuppression is a condition in which the immune response of the body is suppressed due to various reasons resulting in low immunity and increased vulnerability to various diseases. In poultry farming, immunosuppressive diseases in broiler birds are caused by different disorders in the immune responses from the body affecting the abnormal daily feed intake, feed conversion ratio, body weight growth, poor egg production and mortality leading to irreparable economic losses (1–3). In recent years, researchers have carried out a lot of research on the mechanisms and pharmacological control of immunosuppressive diseases in chickens. Since the blind use of antibiotics in poultry and livestock farming has led to development of drug resistance in animals and occurring of different drug residues in food animals. Recently different studies have been planned to focus on the safe and more stable herbs or immunomodulators derived from natural plants (4).

Echinacea was officially used to assist in the treatment of colds, infections, poisonous insects and snake bites in the United States as early as 1887 (5). It has been recorded that Echinacea can proliferate the NK cells of the organism mainly by inhibiting the activity of 5-lipoxygenase and cyclooxygenase (6). It also increases the phagocytic index of granulocytes and enhances the immunity responses of the body *via* production of cytokines such as TNF-α, IFN-β-2, IL-1, and IL-6 by stimulating the macrophages and splenocytes (7, 8).

Astragalus polysaccharides (APS) is an ideal immune promoter that regulates the immune response of different organisms mainly by affecting humoral and cellular immunity. In viral infections APS effectively increases the expression of cytokines such as IL-2, IL-4, IL-6, IL-10, and IL-12 (9). Moreover, it also enhances the phagocytic index of macrophages, stimulates T-cell proliferation and antigen presentation, upregulates levels of immune-related factors, and their ability to synthesize NO by promoting the growth of cells and increasing resistance to viruses (10). APS also has some immunomodulatory capacity. During immune stress, APS reduces transcription of *TLR4* and *NF-*κ*B* genes and inhibits the expression of pro-inflammatory cytokines (11). Furthermore, it affects the morphological development of the jejunum and promotes the synthesis and secretion of specific antibodies following immunization (12). It effectively enhances the efficacy of the vaccine when used as an adjuvant for vaccine during immunization.

The intestinal flora is involved in the digestion and absorption of food, the immune response, growth and development processes, and physiological and structural changes in poultry (13). Research has confirmed that the gut flora is closely linked to host immunity (14) and substance metabolism (15). The causes of lower immunity are different including abnormalities in autoimmunity due to viral or parasitic infections or long-term use or abuse of antibiotics, disruption of the dynamic micro-ecological balance between host and flora, and changes in the number, type, proportion and function of intestinal flora (16). It is recorded that changes in the normal gut flora led to decrease in the productive performance of the organism and even a decrease in immunity, resulting in increased morbidity and mortality, which may cause huge economic losses for the farming industry (17).

The gut microbiota is involved in host immunity in a variety of ways. Most notably through the interactions between immune effector cells and metabolites produced by the gut microbiota during metabolism, which can induce the maturation of immune cells and can activate memory cells. The intestinal epithelium acts as the first barrier for the variety of infectious agents (18) and can possess a wealth of ways of interacting with bacterial metabolites. Metabolites of the intestinal flora are involved in the immune process, dominated by short-chain fatty acids (SCFA), secondary bile acids and tryptophan metabolites (19, 20). Among them, SCFA is a key metabolite in maintaining intestinal homeostasis. It serves as an important source of energy for intestinal epithelial cells and can influence the immune function of the intestinal mucosa by regulating the pH of the intestinal environment (21). The metabolic activity of the microbiota on the intestinal contents has been reported to extensively induce the activation of immune effector cells, thereby enhancing the immune response (22).

Gas chromatograph mass spectrometer (GC-MS) untargeted metabolomics and 16S rDNA intestinal flora sequencing analysis have been used to detect the changes in metabolites and microbiota in the cecum. Therefore, this experimental study was designed and executed to investigate the integrated analysis of the possible effects of Echinacea extract (EE) and APS on the gut microbiota and metabolites of immunosuppressed chickens, and the mechanisms and pathways by which they exert their immunomodulatory effects.

## Materials and methods

### Experimental animals and diets

All the experiments were approved by the Animal Ethics Committee of South China Agricultural University (License Number: 2017A087) and were conducted following the ethical code of conduct for animal care and use. A total of one hundred fast-growing broilers having 4 days of age (male and female) were (purchased from Guangdong Zhiwei Agricultural Technology Co., Ltd.) and were randomly divided into four groups. Both groups C and IS were given ordinary drinking water; group EE: 4 g of EE (purchased from Guangzhou Huanong University Experimental Veterinary Drug Co., Ltd.) per liter of water; group APS: 1.2 g of APS (purchased from Beijing Shengtaier Biotechnology Co., Ltd.) per liter of water. The chickens in all four groups were fed the normal basal diet and fresh water *ad libitum* during the trial.

### Establishment of the immunosuppression model

The birds kept in group C were injected 0.5 mL of normal saline in the pectoral muscle and the birds of other groups (IS, EE, APS) were injected with cyclophosphamide in the pectoral muscle at a dose of 100 mg/kg. Once daily for 3 days.

### 16S-rRNA analysis of gut microbiota

Cecal contents on days 7 and 14 were collected aseptically from the birds and then aliquot at 0.5 g/tube into 2 mL centrifuge tubes. After collection all the samples were immediately frozen in liquid nitrogen and transferred to laboratory and stored at −80°C. Total microbiome DNA was extracted from the gut contents and the target fragments were purified by PCR. Their amplification products were obtained and quantified by fluorescence. On-board libraries were constructed in the TruSeq Nano DNA LT Library Prep Kit, sequenced and the raw data were obtained for further process.

For continued expansion of the sequencing volume, sample size, was first predicted and measured by plotting sparsity and species accumulation curves (23). The top 10 species with the greatest abundance in each group were selected from the taxonomic levels of the phylum and genus based on the results of species annotation. The cumulative bar chart of species relative abundance was generated to visualize the species with greater relative abundance in different taxonomic ranks and their proportions. Alpha diversity reflects the richness of a sample community through metric indices such as Chao1, Shannon, and Simpson. The analysis of beta diversity was performed using multidimensional scalar analysis NMDS. Its results and purposes are to test for similarity and similarity in community structure from different samples (24, 25). RStudio 0.99.447 software (Developed by JJ Allaire Company) was used to count the distribution of sequence lengths contained in the samples, QIIME software (Quantitative Insights Into Microbial Ecology, v1.8.0, http://qiime.org/) (26) was used to classify OTUs, and rare OTUs were removed, and then the identification results were represented by histograms to determine the differences.

### GC-MS metabolomics analysis

A total of 40 mg of intestinal contents was added to 500 μL of pre-cooled water containing 10 μg/mL of demethyl leucine. Samples were sonicated in an ice water bath for 10 min and placed overnight at −20°C. Then the samples were centrifuged at 14,000 g for 15 min at 4°C. Supernatant was removed and extraction of the residue was done. The two supernatants were combined and 100 μL of the supernatant from the extract was obtained and 50 μg/mL norleucine added. Ten microliter of valine was evaporated and 30 μL of methoxypyridine hydrochloride was added to the dry matter and then incubated at 37°C for 90 min. After that 30 μL of BSTFA containing 1% TMCS was added, vortexed for 30 s, and then derivatized. Detection and analysis were performed using a gas chromatograph-mass spectrometer. The raw GC-MS data were processed using excel with reference to literature methods (27). The raw data from GC-MS was automatically deconvoluted using AMDIS software and matched against a self-built standard database (including retention times and mass spectra), the Golm metabolome database, and the Agilent Fiehn GC/MS metabolomics RTL library.

The data files were imported into SIMCA software (version 14.1) for multidimensional statistical analysis such as PCA and PLS-DA. The data were first formatted with the default mean-centered and UV (unit variance) before analysis. Then the optimal principal component scores were automatically calculated and the optimal model was built. To avoid model overfitting, the optimal number of principal components is calculated using the default 7-round cross-validation (7-round cross-validation) of SIMCA software. The model quality evaluation parameters are R2X or R2Y and Q2 values, which are used to evaluate the robustness of the pattern recognition model, where R2X (PCA) or R2Y (PLS-DA) indicates the proportion of data variance that can be explained by the current model, i.e., the explanation rate, which indicates the goodness of fit of the model. Q2 indicates the proportion of data variance that can be predicted by the current model, i.e., the prediction rate, which indicates the predictive power of the current model.

The combination of a VIP value (Variable importance in the projection) >1 and a *p*-value <0.05 for unidimensional statistical analysis was used to screen for differential metabolites. Fold change (Log2FC) was calculated as the logarithm (with a base of 2) of the ratio of the mean values of the data from group 1 and group 2, with a positive value indicating that the substance was at a higher level in group 1 than in group 2 and a negative value indicating the opposite.

### Analysis of the link between flora and metabolic processes

After sequencing and analysis of the intestinal flora, flora with significant differences were selected. The metabolites of these flora were collected through literature review and database searches. Using the online mapping software Bioladder, the data for the differential metabolites were transformed into volcano plots for display, and metabolites with significant differences were identified in the plots using *P* < 0.05 and Log2FC absolute values ≥1 as screening criteria. Pathway analysis of the differential metabolites was performed using the online software MetaboAnalyst (version 4.0 http://www.metaboanalyst.ca/) according to the metabolites with significant differences. The results of both were correlated to analyze the relationship between the gut microbiota and metabolic activity in the gut.

## Results

### Diversity analysis

**Figure 1:**
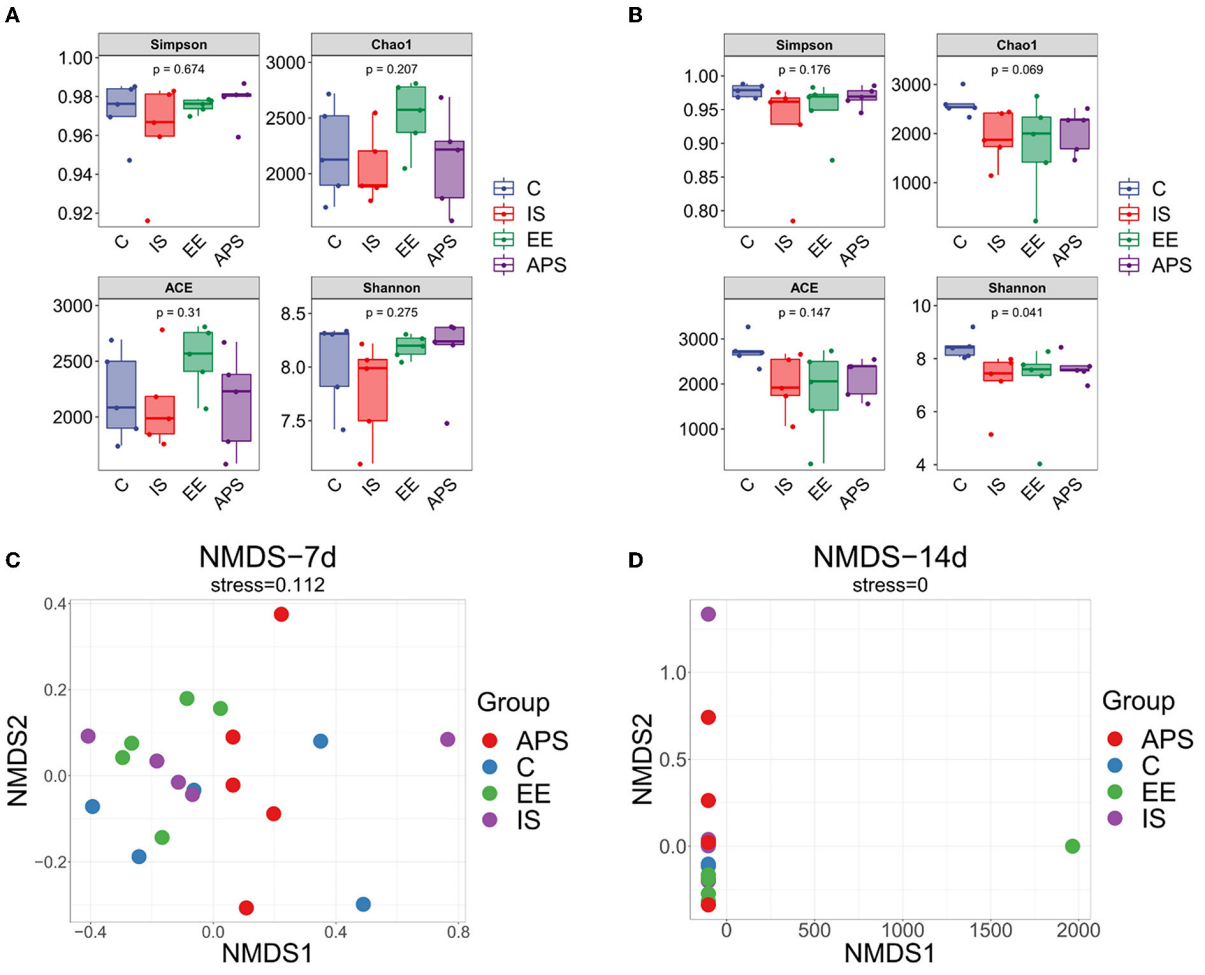
Diversity analysis result. Alpha diversity index difference box plot **(A)** 7d; **(B)** 14d. NMDS analysis plots **(C)** 7d; **(D)** 14d (*N* = 5).

The results on diversity analysis showed that the number of OTUs in the broilers gut microbiota tested in the samples were very rich. The OTU division and classification status also differed between the groups. At day 7 of the trial, no significant differences were found in the α-diversity indices of Ace, Chao1, Shannon, and Simpson (Figure 1A, *P* > 0.05). On day 14 of trial, except for the Shannon index (*P* < 0.05), there was no significant difference in other indices (Figure 1B, *P* > 0.05). Results showed that under the influence of immunosuppression, the species community diversity of the gut microbiota was affected. The samples were significantly dispersed (Figure 1C) but the groups gradually approached over time (Figure 1D) after the cessation of immunosuppression. The results showed that the impact of immunosuppression on the gut microbiota of chickens could be gradually alleviated over time, and the species community diversity in each group showed a recovery trend, among which the species abundance diversity recovered more significantly.

### Statistics on the number of microbial taxa at each taxonomic level

**Figure 2:**
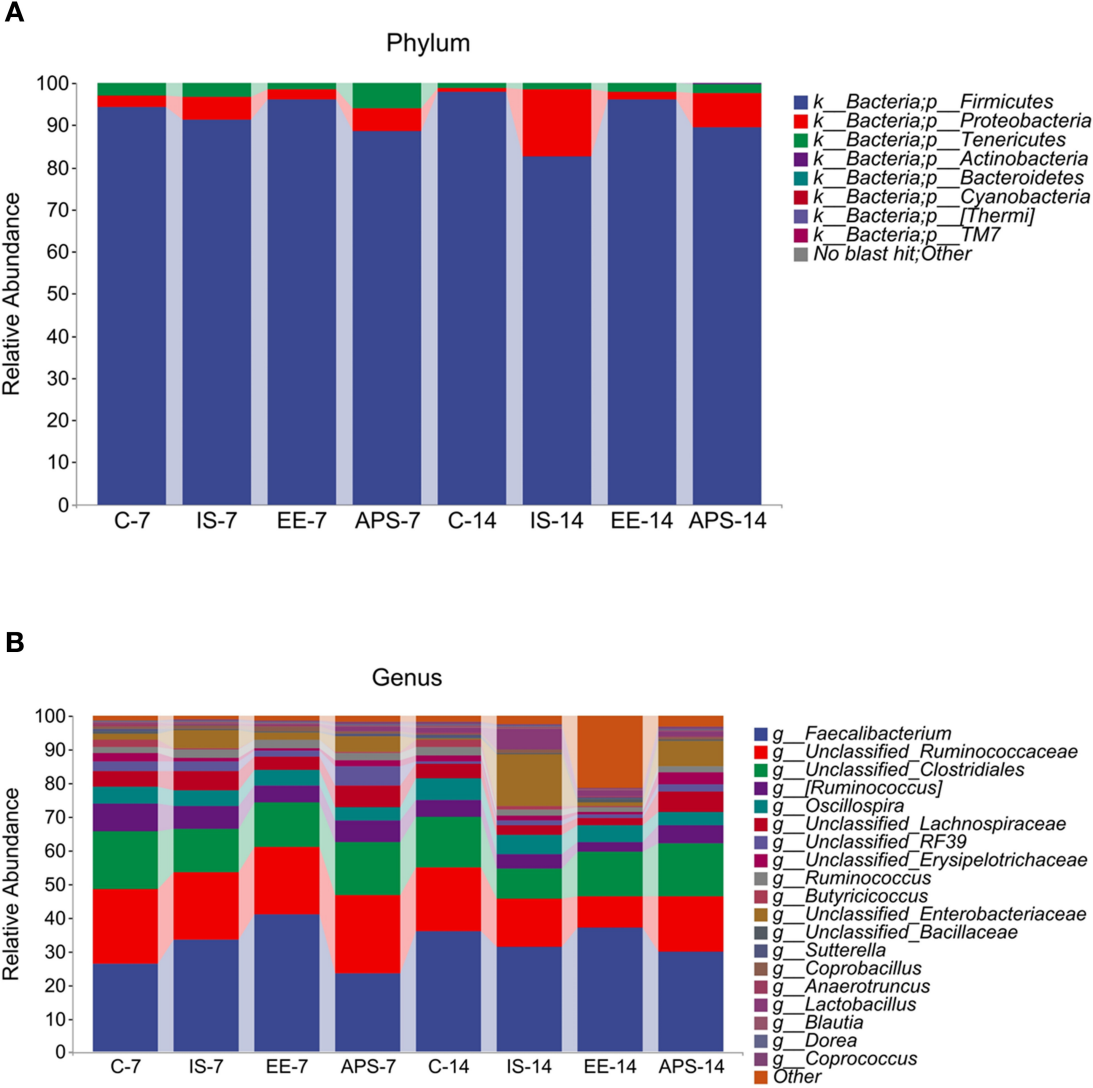
Map of taxonomic composition and abundance distribution of communities at the phylum and genus level. **(A)** Phylum; **(B)** Genus. The horizontal coordinates are arranged according to groups at different times with each bar representing a group and colors distinguishing each taxonomic unit. The vertical coordinates represent the relative abundance of each taxonomic unit. The longer the bar, the higher the relative abundance of that taxonomic unit in the corresponding sample (*N* = 5).

The taxonomic test results showed that six major taxa were detected at the phylum level, including *Firmicutes, Proteobacteria, Tenericutes, Bacteroidetes, Actinobacteria* and *Cyanobacteria* (Figure 2A), while at the genus level for *Faecalibacterium, Oscillospira, Ruminococcus, Lactobacillus, Mollicutes_RF39, Enterococcus, Anaerotruncus*, and *Sutterella* (Figure 2B). Over the course of the experiment, the relative abundance of Firmicutes was similar in group C (94.37% at 7 days and 97.83% at 14 days) and group EE (95.98% at 7 days and 96.22% at 14 days), which had a higher percentage of Firmicutes than the IS and APS groups. The IS group reduced the Firmicutes. The IS group decreased the relative abundance of Firmicutes from 91.29 to 82.65% in the experiment, while all other groups showed an increasing trend. In contrast, the relative abundance of Proteobacteria was higher in the IS group, increasing from 5.43 to 15.75%, and similarly, the APS group showed an increasing trend (from 5.51 to 8.19%). The other two groups showed a decreasing trend, accounting for only 1.08% (C) and 1.66% (EE) at 14 days (Figure 2A). At the genus level, the relative abundance of Enterobacteriaceae changed significantly in the IS group, increasing from 5.37 to 15.48%, which was significantly higher than the content share of other groups. At the genus level, the relative abundance of Enterobacteriaceae varied significantly in the IS group, increasing from 5.37 to 15.48%, which was remarkably higher than the content share of the other groups, of which the APS group showed an increasing trend (4.91–7.44%). On the contrary, the other two groups showed a decreasing trend (Figure 2B).

### Analysis of differences in taxonomic composition between groups

**Figure 3:**
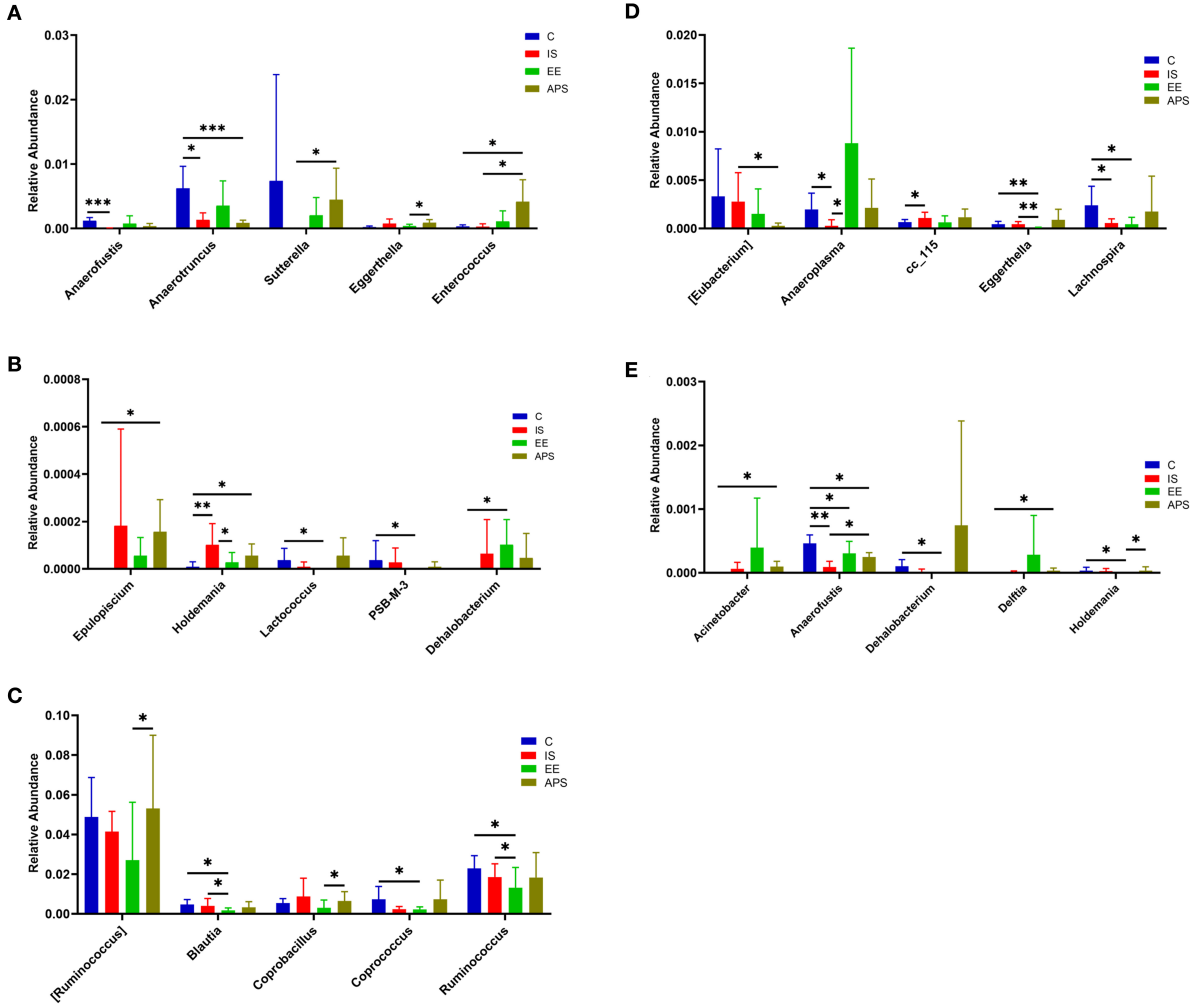
**(A,B)** Represent the abundance distribution of the top 10 taxa with the most significant differences between groups at 7 days; **(C–E)** represent the top 15 taxa with the most significant differences at 14 days Abundance distribution of the taxa (*P < 0.05; **P < 0.01; ***P < 0.001) (*N* = 5).

After 7 days, compared with group C, the abundance of *Anaerotruncus* (*P* < 0.05) and *Anaerofustis* (*P* < 0.001) in IS group were significantly decreased, and *Holdemania* was significantly increased (*P* < 0.01). Compared with IS group, *Holdemania* in EE group was significantly decreased (*P* < 0.05); compared with IS group, *Enterococcus* and *Sutterella* were significantly increased in APS group (*P* < 0.05; Figures 3A,B; Table 1). At day 14 of the trial, compared with the C group, the abundance of *Anaerofustis* (*P* < 0.01), *Anaeroplasma* (*P* < 0.05) and *Lachnospira* (*P* < 0.05) in the IS group was significantly reduced, and *cc_115* was significantly increased (*P* < 0.05); Compared with the IS group, the *Anaeroplasma* in the EE group was significantly increased (*P* < 0.05). Compared with the IS group, the *Anaerofustis* (*P* < 0.05) in the EE group was significantly increased, and *[Eubactreium]* (*P* < 0.05) was significantly less in the IS group (Figures 3C–E; Table 1).

**Table 1:** Summary of genera with significant variation during the trial.

**Genus**	**IS vs. C**	**EE vs. IS**	**APS vs. IS**
*Anaerofustis*	↓^***^	↑	↑^*^
*Anaeroplasma*	↓^*^	↑^*^	↑
*Anaerotruncus*	↓^*^	↑	↓
*Blautia*	↓	↓^*^	↓
*cc_115*	↑^*^	↓	-
*Eggerthella*	↑	↓^**^	↑
*Enterococcus*	-	↑	↑^*^
*Holdemania*	↑^**^	↓^*^	↓
*Lachnospira*	↓^*^	-	↑
*Ruminococcus*	↓	↓^*^	↑

The table is not divided by time periods and only based on the results of the trial process.

“↑” indicates that the abundance of the genus increases, “↓” indicates a decrease, and “-” indicates that it cannot be determined.

* *P* <0.05.

** *P* <0.01.

*** *P* <0.001.

### Principal components analysis

Principal component analysis was performed on the intestinal contents of each group at day 7 and the cumulative interpretation rate of the model was R^2^X = 0.585. Current PCA models can be reliably used to describe metabolic differences between samples. There was separation between group C and the other three groups.

Principal component analysis was performed on the intestinal contents of each group at 14 days, and the cumulative interpretation rate of the model was R^2^X = 0.604. Current PCA models can be reliably used to describe metabolic differences between samples. There was a significant separation between group C and the other three groups.

### Partial least squares discrimination analysis

A PLS-DA model with 2 effective principal components was developed for the content at day 7 in treatment group, R^2^X = 0.338, R^2^Y = 0.445, and *Q*^2^ = 0.174, and the PLS-DA score plot showed a significant separation between at day 7 in group C and the other 3 sample groups. A PLS-DA model with 2 effective principal components was developed at day 14 of treatment group of contents, R^2^X = 0.288, R^2^Y = 0.527, and *Q*^2^ = 0.268, and the PLS-DA score plot showed a trend toward separation between the four sample groups.

### Differential analysis of metabolites and metabolic pathways

**Figure 4:**
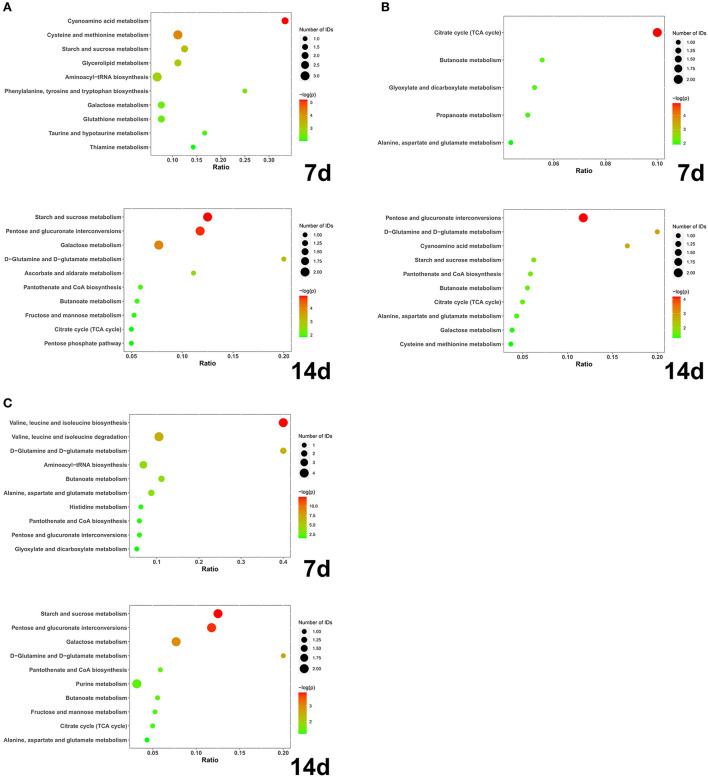
**(A)** IS-C; **(B)** EE-IS; **(C)** APS-IS. The abscissa axis Ratio represents the ratio of differential metabolites enriched to this metabolic pathway to total metabolites; the size of bubbles represents the number of differential metabolites enriched to this metabolic pathway; the larger the -log(*P*), the smaller the *P*-value and the more significant the difference (*N* = 5).

At day 7, a total of 28 different substances were screened (Table 2). At 14 days, a total of 22 different substances were screened (Table 3). Compared with group C, the different metabolic pathways of intestinal contents in group IS at day 7 were Cyanoamino acid metabolism, Cysteine and methionine metabolism, Starch and sucrose metabolism, and Glycerolipid metabolism. At 14 days, it is Starch and sucrose metabolism, Pentose and glucuronate interconversions, Galactose metabolism, D-Glutamine and D-glutamate metabolism, Ascorbate and aldarate metabolism, Pantothenate and CoA biosynthesis, Butanoate (Figure 4A). At 7 days, compared with IS, the different metabolic pathways of intestinal contents in EE were Citrate cycle (TCA cycle), Butanoate metabolism, Glyoxylate and dicarboxylate metabolism, Propanoate metabolism, Alanine, aspartate and glutamate metabolism. At 14 days, it is Pentose and glucuronate interconversions, D-Glutamine and D-glutamate metabolism, Cyanoamino acid metabolism (Figure 4B). Compared with IS, the differential metabolic pathways of intestinal contents in APS at 7 days are Valine, leucine and isoleucine biosynthesis, Valine, leucine and isoleucine degradation, D-Glutamine and D-glutamate metabolism, Aminoacyl-tRNA biosynthesis, Butanoate metabolism, Alanine, aspartate and glutamate metabolism. At 14 days, it is Starch and sucrose metabolism, Pentose and glucuronate interconversions, Galactose metabolism, D-Glutamine and D-glutamate metabolism (Figure 4C).

**Table 2:** Differential metabolites of intestinal contents in each group at 7 days (|Log[2]FC| ≥ 1).

	**Metabolites**	**VIP**	***p*-value**	**Log_2_FC**	**HMDB**	**KEGG**
IS-C	Maltose	1.46	1.46E-02	−1.98	HMDB0000163	C00208
	3-Cyanoalanine	1.74	3.66E-04	−1.95	METPA0300	C02512
	Glutaric acid	1.32	2.91E-02	−1.94	HMDB0000661	C00489
	3-Hydroxybenzoic acid	1.35	2.71E-02	−1.72	HMDB0002466	C00587
	Glutamine[-H_2_O]	1.78	1.34E-04	−1.60	-	-
	4-Hydroxybenzoic acid	1.49	9.01E-03	−1.58	HMDB0000500	C00156
	4-Hydroxybutyric acid	1.33	3.27E-02	−1.13	HMDB0000710	C00989
	2-Deoxyinosine	1.36	1.93E-02	1.29	HMDB0000071	C05512
	Galactose	1.50	1.11E-02	1.32	HMDB0000143	C00984
	2-Deoxyguanosine	1.32	3.02E-02	1.35	HMDB0000085	C00330
	Glycerol-3-phosphate	1.22	3.43E-02	1.44	HMDB0000126	C00093
	Cystine	1.42	1.49E-02	1.48	HMDB0000192	C00491
	Orotic acid	1.49	1.50E-02	3.11	HMDB0000226	C00295
	Lactose	1.53	1.27E-02	3.22	HMDB0000186	C00243
EE-IS	Succinic acid	1.48	2.72E-02	−1.29	HMDB0000254	C00042
	Citric acid	1.60	2.00E-02	1.02	HMDB0000094	C00158
	Hexanoic acid	1.86	3.67E-03	1.37	HMDB0000535	C01585
	4-Methylvaleric acid	1.64	2.83E-02	1.63	HMDB0000689	-
	3-Hydroxyphenylacetic acid	1.94	9.78E-04	1.93	HMDB0000440	C05593
	Glutaric acid	1.98	1.39E-03	2.04	HMDB0000661	C00489
APS-IS	3,4-Dihydroxyphenylacetic acid	1.62	1.20E-02	−2.49	HMDB0001336	C01161
	Malic acid	1.42	4.97E-02	1.00	HMDB0000156	C00149
	4-Hydroxyphenylethanol	1.78	2.67E-03	1.01	HMDB0004284	C06044
	2-Hydroxyglutaric acid	1.40	4.71E-02	1.22	HMDB0000694	C03196
	4-Methylthio-2-ketobutyric acid	1.88	3.77E-04	1.50	HMDB0001553	C01180
	2-Ketoglutaric acid	1.68	6.60E-03	1.73	HMDB0000208	C00026
	2-Ketoisovaleric acid	1.74	3.75E-03	1.84	HMDB0000019	C00141
	3-Methyl-2-ketovaleric acid	1.81	1.54E-03	1.88	HMDB0000491	C03465
	2-Ketoisocaproic acid	1.87	5.55E-04	1.88	HMDB0000695	C00233

The underlined value means that the number has corresponding information in the database.

**Table 3:** Differential metabolites of intestinal contents in each group at 14 days (|Log[2]FC| ≥1).

	**Metabolites**	**VIP**	***p*-value**	**Log2FC**	**HMDB**	**KEGG**
IS-C	2-Ketoglutaric acid	1.39	4.11E-02	−1.61	HMDB0000208	C00026
	Beta-glutamic acid	1.50	1.77E-02	−1.35	-	-
	4-Hydroxyphenylacetic acid	1.56	3.52E-02	1.00	HMDB0000020	C00642
	Fructofuranose	1.65	9.37E-03	1.12	-	-
	Gluconic acid	1.59	2.38E-02	1.49	HMDB0000625	C00257
	Glycyl-leucine	1.30	4.13E-02	1.63	HMDB0000759	C02155
EE-IS	2-Hydroxyglutaric acid	1.64	9.38E-03	−1.43	HMDB0000694	C03196
	2-Ketoglutaric acid	1.59	1.55E-02	−1.27	HMDB0000208	C00026
	4-Hydroxyphenylacetic acid	1.54	2.50E-02	−1.01	HMDB0000020	C00642
	3-Cyanoalanine	1.46	3.93E-02	−1.00	METPA0300	C02512
	Ethanolamine	1.52	2.50E-02	1.28	HMDB0000149	C00189
	5-Hydroxyindoleacetic acid	1.64	9.80E-03	1.43	HMDB0000763	C05635
	Coprostanol	1.47	3.46E-02	2.25	HMDB0000577	-
APS-IS	3-Hydroxybenzoic acid	1.48	1.43E-02	−2.77	HMDB0002466	C00587
	Fructose	1.74	1.86E-03	−1.59	HMDB0000660	C02336
	2,3-Dihydroxybutane	1.47	2.80E-02	−1.52	HMDB0003156	C00265
	Xylitol	1.47	1.34E-02	−1.25	HMDB0002917	C00379
	Glucose	1.57	1.50E-02	−1.22	HMDB0000122	C00031
	Fructofuranose	1.51	4.08E-02	−1.17	-	-
	Adenine	1.44	4.52E-02	1.13	HMDB0000034	C00147
	2-Deoxyadenosine	1.34	4.00E-02	1.15	HMDB0000101	C00559
	Stigmastanol	1.59	1.46E-02	1.40	HMDB0000494	-
	Octanoic acid	1.51	1.85E-02	1.68	HMDB0000482	C06423
	p-cresol	1.43	3.28E-02	1.68	HMDB0001858	C01468
	2-Ketoglutaric acid	1.55	1.91E-02	1.81	HMDB0000208	C00026
	Uric acid	1.44	2.05E-02	1.85	HMDB0000289	C00366

The underlined value means that the number has corresponding information in the database.

## Discussion

There was found that the species of bacteria in the caecum of laying hens varies with age and does not become stable until 12–16 weeks (28). The changing trend of intestinal flora may be similar during the growth and development of broilers and laying hens. Throughout the experiment, broiler birds grew from day 1–4 weeks of age possibly with changes in gut microbiota species occurring in this period. The effects of immunosuppression on the gut microbiota may coincide with changes in microbiota species. A tendency for the microbiota of each group to approach each other over time was observed in the NMDS analysis, and changes in gut microbiota species with age may be a factor in our study. In addition, the diversity of microbiota abundance was restored with the gradual recovery of immune functions with the cessation of immunosuppression. Nevertheless, in this process, there were no significant differences in the alpha diversity index except for the Shannon index. This trend of variation revealed that the process of immunosuppression had no significant effects on the number of species in the microbiota, but produced a certain degree of variation in the abundance of species in the microbiota.

Ten different genera were identified by differential analysis of the gut microbiota. *Anaerofustis, Anaeroplasma, Anaerotruncus, Blautia, cc_115, Eggerthella, Enterococcus, Holdemania, Lachnospira*, and *Ruminococcus*, respectively. Among them, *Anaerofustis, Anaeroplasma, Anaerotruncus, Lachnospira*, and *Ruminococcus* belong to *Firmicutes*. They often produce short-chain fatty acids (SCFAs) such as butyric acid and propionic acid directly or indirectly (29–33). SCFAs has anti-inflammatory, anti-tumor, anti-bacterial and immunomodulatory functions. This type of metabolic derivative achieves an anti-inflammatory effects by inhibiting the proliferation of phagocytes and reducing the levels of inflammatory factors, thereby blocking the activation of the NF-κB pathways (30) which builds chemical and physical barriers in the gut and assists in intestinal immune balance.

The intestinal abundance of the above microbiota was significantly reduced in the immunosuppressed state, with the exception of *Ruminantococcus*. Under this condition, it may affect the metabolic production of SCFAs and increase the risk to intestinal health. Instead, the two groups treated with EE and APS promoted the proliferation of colonies reduced in abundance by immunosuppression and increased their abundance, including *Ruminococcus*. In addition, an increased abundance of *Enterococcus* was found in both groups. *Enterococcus* was once considered to be a threatening pathogen. It has been found to be clinically resistant in human medicine, often causing bacteraemia and death (34). But recent research has found that *Enterococcus* has a protective role in the gut. Feeding *Enterococcus* to laying hens by mixing it into their diets could reduce damage to the intestinal mucosa during Salmonella infection. It enhances the immune functions of the organism by reducing oxidative stress and down-regulating serum malondialdehyde levels, while up-regulating IgG levels (35). The increase in the relative abundance of Enterococcus in the gut can protect the gut and have a positive effects on the immunity of the organism. The above evidence indicates that both EE and APS can regulate the immunity by increasing the relative abundance of beneficial bacteria in the gut, and relieve the organism's immune-suppressed state.

The relative abundance of *Eggerthella* was increased in the gut of immunosuppressed chickens. The relevance of *E. lenta* to autoimmune diseases in humans has been confirmed by research (36). It may induce the activation of Th17 cells through its microbial metabolism, contributing to an excessive inflammatory response and exacerbating symptoms in mice with colitis (22). Studies have shown that *E. lenta* can enhance host autoimmunity through the activation of helper T cells. The increased relative abundance of this genus may be associated with suppression of the overall immune functions of the host.

EE is a more targeted modulation of the organism's immune function than APS. EE significantly reduced the relative abundance of *Eggerthella, Holdemania* in the gut of immunosuppressed chickens but was not observed in APS group. Additionally, in the EE group, the relative abundances of *Blautia* and *Ruminococcus* were found to be down-regulated. The relative abundance of *Blautia* in the gut is positively correlated with age (37, 38) and immune inflammatory response (39). It also correlates significantly with host physiological functions, such as obesity metabolism (40) and cancer. It has been reported that its biochemical metabolism can produce some carcinogens such as secondary bile acids, carbolic acid and deoxycholic acid (41). EE down-regulated its relative abundance in the gut, possibly revealing that EE plays a bidirectional regulatory role in regulating immune status in chickens.

Metabolomic analysis of intestinal contents revealed that EE and APS may influence butyrate metabolism, alanine, aspartate and glutamate metabolism, the interaction of pentose and glucuronide conversion, and D-glutamine and D-glutamate metabolism. Both may regulate the organism's gut immune functions through their effects on intestinal metabolic processes. This influence is reflected in the metabolic pathways of butyrate metabolism, alanine, aspartate and glutamate metabolism, with both butyrate and aspartate having a positive effect on intestinal immunity. For example, butyric acid, a member of the SCFAs, provides energy to the intestinal cells and maintains the integrity of the intestinal barrier. It also regulates the balance of the gut microbiota by acidifying the intestinal environment. Butyric acid has been shown to affect intestinal immune function by inhibiting the migration of immune cells and regulating cell proliferation and apoptosis (42–44). Together with glutamic acid, asparagine acts as precursors for the synthesis of various amino acids. They provide energy to the intestine, protect the intestinal mucosa, reduce mucosal damage caused by bacterial endotoxins (45), delay lymphocyte apoptosis and promote cell growth.

To reveal the effects of EE and APS on the gut of immunosuppressed chickens by correlating gut microbiota and content metabolomics. Not only is the gut involved in changes in the entire intestinal environment, but also the microbiota within it. The metabolites of the intestinal flora play an important role in the physiological processes of the organism, protecting intestinal health (46), providing energy, activating immune cells, participating in the intestinal immune process and regulating the balance of the intestinal flora. The succinate producing bacteria *Blautia* and *Ruminococcus* were discovered to be associated with some differential metabolic pathways in the metabolomic analysis. Among these were alanine, aspartate and glutamate metabolism, butyrate metabolism, TCA cycle, glyoxylate and dicarboxylic acid metabolism and propionic acid metabolism. Succinate, an important signaling factor for immune activation, has a key role in the regulation of inflammation (47, 48) and a role in sustaining the stability of the intestinal lining in the gut (49). Elevated levels of succinate in the gut may promote intestinal inflammation (50). EE might have a reverse immunomodulatory effects by reducing the amount of succinic acid in the gut, affecting the energy metabolism of glucose metabolism, the metabolism of butyric and propionic acids and the metabolism of immune-related amino acids based on the results of the trial. But its more precise mechanism of action and related effects needs more experimental studies to verify the findings.

## Conclusion

This trial used 16S-rRNA analysis of the gut microbiota and metabolomics to analyze the effects of EE and APS on immunosuppressed chickens. Overall, immunosuppression can affect the species diversity of the intestinal flora and reduce the relative abundance of some SCFAs-producing bacteria in the gut. Both EE and APS can play a role in mitigating the effects of immunosuppression on the intestinal flora, restoring the abundance of beneficial bacteria and anaerobic genera, and protecting the health of the intestinal environment. The comprehensive analysis of the metabolism of the microbiota revealed that EE might suppress the relative abundance of *Blautia* and *Ruminococcus*, reducing the succinic acid content in the intestine.

## Data availability statement

The raw data supporting the conclusions of this article will be made available by the authors, without undue reservation.

## Ethics statement

The animal study was reviewed and approved by the Animal Ethics Committee of South China Agricultural University (License Number: 2017A087). Written informed consent was obtained from the owners for the participation of their animals in this study.

## Author contributions

SL, RL, JC, SZ, and YS were responsible for study conception and design. RH, DS, and AG revised the manuscript. SL, RL, JC, SZ, YS, and DS were involved in the drafting of the manuscript. All authors contributed to the article and approved the submitted version.

## Funding

This study was supported by General Program of Natural Science Foundation of Guangdong Province (2021A1515011010) and Key R&D Projects in Guangzhou (202206010189).

## Conflict of interest

The authors declare that the research was conducted in the absence of any commercial or financial relationships that could be construed as a potential conflict of interest.

## Publisher's note

All claims expressed in this article are solely those of the authors and do not necessarily represent those of their affiliated organizations, or those of the publisher, the editors and the reviewers. Any product that may be evaluated in this article, or claim that may be made by its manufacturer, is not guaranteed or endorsed by the publisher.
